# Mask wearing increases eye involvement during smiling: a facial EMG study

**DOI:** 10.1038/s41598-021-99872-y

**Published:** 2021-10-13

**Authors:** Shuntaro Okazaki, Haruna Yamanami, Fumika Nakagawa, Nozomi Takuwa, Keith James Kawabata Duncan

**Affiliations:** MIRAI Technology Institute, Shiseido Co. Ltd., 1-2-11 Takashima, Nishi-ku, Yokohama, Kanagawa 220-0011 Japan

**Keywords:** Psychology, Human behaviour

## Abstract

The use of face masks has become ubiquitous. Although mask wearing is a convenient way to reduce the spread of disease, it is important to know how the mask affects our communication via facial expression. For example, when we are wearing the mask and meet a friend, are our facial expressions different compared to when we are not? We investigated the effect of face mask wearing on facial expression, including the area around the eyes. We measured surface electromyography from zygomaticus major, orbicularis oculi, and depressor anguli oris muscles, when people smiled and talked with or without a mask. Only the actions of the orbicularis oculi were facilitated by wearing the mask. We thus concluded that mask wearing may increase the recruitment of the eyes during smiling. In other words, we can express joy and happiness even when wearing a face mask.

## Introduction

How does the use of a face mask influence our daily communication? Although a part of Japanese culture since the Meiji Era (1868–1912)^1^, face mask usage has only recently become ubiquitous across the world during the outbreak of severe acute respiratory syndrome coronavirus 2 (SARS-CoV-2), the virus that causes coronavirus disease 2019 (COVID-19). Unsurprisingly, interest has grown in the physiological and psychological effects of mask wearing^2^, especially as there are concerns regarding the potential interference with non-verbal communication, which may contribute to resistance to mask wearing.

Facial expression is one of the most efficient channels to communicate our feelings to others^3^. Among these expressions, smiling is a fundamental facial expression to communicate positive affect. A smile results when the corners of the mouth are raised by the contraction of zygomaticus major. It strongly influences the attention, the brain activity, and various behaviors in others^4^. As the mask hides the mouth, it is perhaps unsurprising that recent studies have argued that facial mask wearing interferes with the recognition of the wearer’s emotion and impression^5,6^.

However, a smile can also include contraction of orbicularis oculi, which raises the cheeks and creases the skin at the corners of the eyes. When it does, it is referred to as a Duchenne smile^7,8^. Relative to smiles without eye muscle involvement, Duchenne smiles have a stronger effect on the evaluation of happiness^9^ and desirable impression^10,11^ by a viewer. The importance of eye involvement is underscored by the finding that botulinum toxin injection into orbicularis oculi diminishes eye involvement in smiling and decreases the perceived happiness of the person who received the treatment^12^. The hiding of the mouth by the mask increases the importance of the upper face in emotional expression^3,13^. However, it is unknown if individuals adapt the use of their upper face muscles to compensate for wearing a mask. We hypothesized that wearing a mask will enhance eye muscle involvement in smiling as the mask wearer attempts to communicate their smile to others.

To investigate the effect of wearing a mask on facial expression, we used a sequence of tasks with concurrent facial EMG recording of orbicularis oculi, zygomaticus major, and depressor anguli oris. Orbicularis oculi and zygomaticus major were chosen due to their involvement in smiling and depressor anguli oris chosen because of its involvement in speech^14^. There are 4 possibilities. First, if our hypothesis is correct and the participants try to overcome the mask interference by selectively increasing the eye muscle involvement, only orbicularis oculi activity will be enhanced when smiling with the mask. Second, if the mask disturbs facial expression, but the individual control of the smile related muscles is difficult, both orbicularis oculi and zygomaticus major will be enhanced when smiling with the mask. Third, if the mask disturbs general facial motion physically, the mask wearer may universally exaggerate all their facial motions, enhancing both smiling (orbicularis oculi, zygomaticus major) and speaking (depressor anguli oris) to resist the difficulty in the motion. Finally, if the mask does not influence facial motion, there will be no change in any muscles.

## Materials and methods

### Participants

Twenty female participants (39.8 ± 11.9 years old) received remuneration for participation in this experiment, after providing their written informed consent. They reported normal or corrected-to-normal vision and no history of psychiatric and neurological disorders. This study was performed in accordance with the principles in the Declaration of Helsinki and approved by the Ethical committee at the Shiseido Global Innovation Center (Approval number: C02121).

### Stimuli and procedure

**Figure 1:**
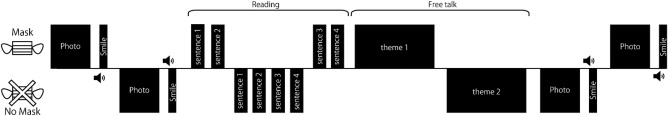
Schematic representation of the experimental design.

The participants sat comfortably on a chair and were asked to perform 4 behavioural tasks according to the instructions that appeared on the PC screen in front of them (Fig. 1). The first task was the “photo” task. In this task, the participants watched a movie in which an experimenter appeared and pretended to take a picture of the participant, who was asked to smile. The second was the “smiling” task. Participants were asked to make their biggest smile (pull the mouth corner back maximally with the choice of whether to open their mouth or not left to them) immediately after they heard a beep from the PC. The third was the “reading” task. The participants read aloud the designated 4 ATR 503 phonetically balanced sentences that were displayed on the screen. The fourth task was the “talking” task. The participants talked about 2 themes (“What I’m addicted to these days” and “What I have enjoyed recently”) as long as they could, for up to a maximum of 30 s. They were also asked to pretend to talk with a person familiar to them in this task.

The photo and smiling tasks were both conducted twice (once at the beginning and once at the end of the experiment) for each mask condition. All tasks were implemented with or without a surgical mask on the face (Fig. 1). The order of mask condition (with or without mask) was counterbalanced by the repetition (once at the beginning and once at the end of the experiment) and across participants. After the experiment was started, the experimenter left the participants alone in the experimental room as an anti-coronavirus infection risk countermeasure and let them initiate the start of the experiment by themselves which they did by pushing the spacebar of the PC. During this time, the participant was recorded on video. Detailed procedures of the behavioural tasks and timing of wearing and taking off the mask were instructed by the in-house developed Python 3.7.3 (Python software foundation, https://www.python.org/) program made with the PsychoPy library^15^.

### Data acquisition and analysis

Three active bipolar Ag electrodes (AP-C140-020, Miyuki Giken Co., Ltd., Tokyo, Japan) were attached on the face to acquire the EMG from the orbicularis oculi and zygomaticus major muscles on the left, and from the depressor anguli oris muscle on the right. The detailed positioning was decided in accordance with published guidelines^16^. Reference and Grand electrodes (AP-C151-015 and MA-C004-015 with general disposable electrode, respectively) were both attached on the forehead and covered with a hairband. To lower the electrode conductance, we applied Ten20 conductive paste (Weaver and Company, Aurora, CO, USA) on the electrode tips and attached a pierced double-sided tape between the body of the electrode and skin. They were also covered with elastic tape.

EMG signals were recorded by a 32-ch physiological signal recording device (Polymate V AP5148, Miyuki Giken Co., Ltd., Tokyo, Japan) with a sampling rate of 1000 Hz. 50-Hz noise in the EMG was excluded by a notch filter (48–52 Hz). Then spiky outlier data over 200 mV was removed. The filtered signal was rectified and integrated within each 20-ms time window^16^ to monitor its time series. The preprocessed EMG signals were divided into epochs for each behavioural task based on the audio signals (beep for the smiling task and voice onset for the photo, reading, and talking tasks). Due to the self-proceeding experimental procedure, the epochs where the participants failed the procedures were manually checked on the recorded video and excluded from further analyses. This process was done by a rater who took no further role in the data analysis. These failures included failure to comply with the task (e.g. due to failure to hear instructions), misspeaking or misreading. In the photo and smiling tasks, if both trials were rated as successful, the signals from the two trials were averaged. If only one trial was rated as a success and one as a fail, only the successful trial was used in further analysis. The EMG signal was averaged during the tasks and compared between conditions with or without the mask by using a Wilcoxon signed-rank test with a Bonferroni correction (N = 12 for tasks and measured muscles). Signed-rank (V), sample size (n), corrected (or uncorrected) *p* value, and effect size (r = Z/√n) were shown as statistics.

## Results

**Figure 2:**
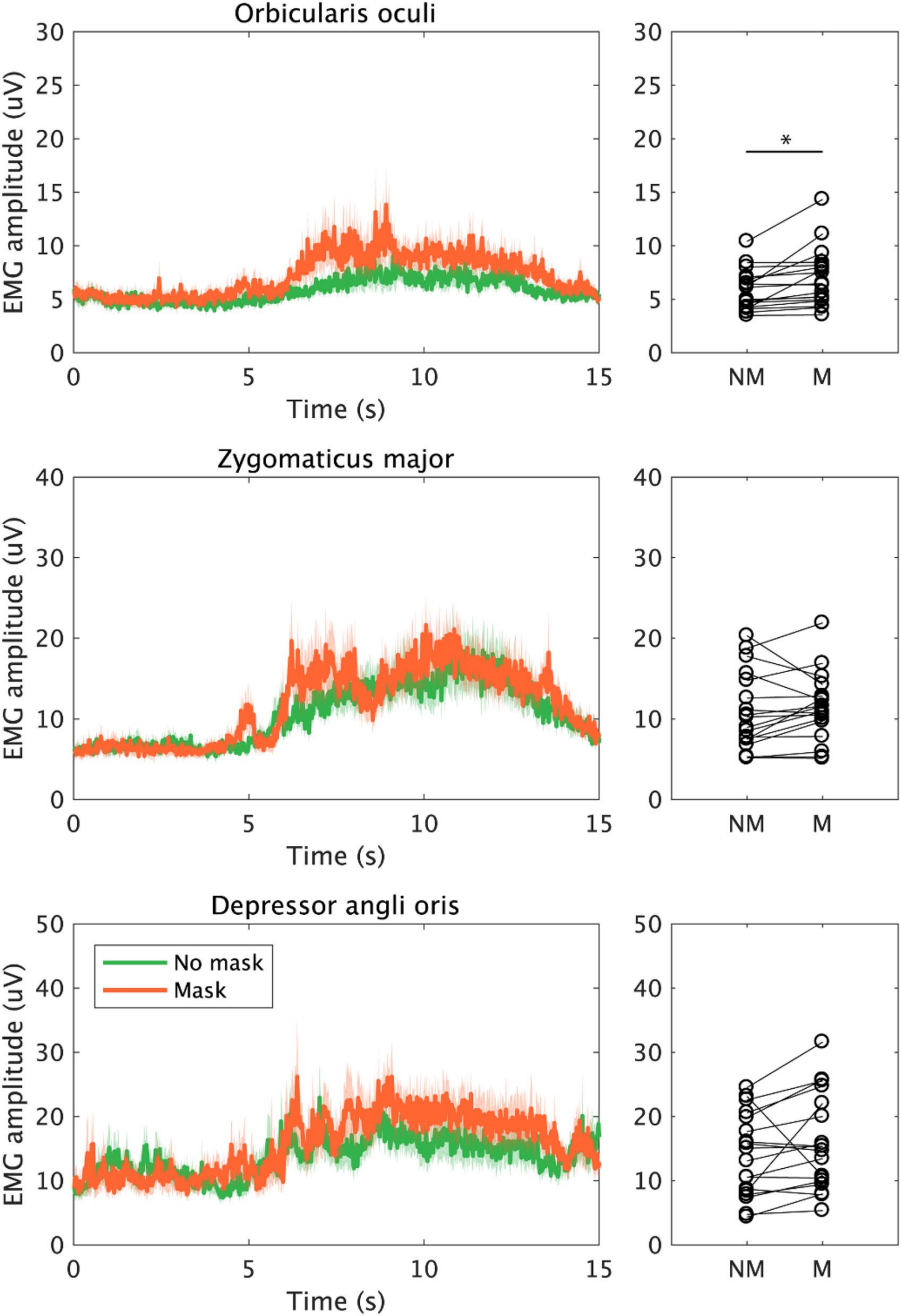
EMG signal measured over orbicularis oculi, zygomaticus major, and depressor anguli oris, during the photo task. The graphs in the left column indicate the time series of EMG signal of these muscles that were integrated with each 20-ms time window (sampling rate was 50 Hz). The solid line is the averaged EMG and the coloured area indicates a standard error of the mean (SEM) across participants. Orange and green lines indicate the EMG signal when the participants wore the mask and when they did not, respectively. Right columns indicate comparisons of averaged EMG during the whole task (15 s) when the participants wore the mask (M) and when they did not (NM). An asterisk indicates a significant difference in the averaged EMG between NM and M conditions (Wilcoxon signed-rank test with a Bonferroni correction).

The photo task average EMG signals recorded on the 3 muscles (orbicularis oculi, zygomaticus major, and depressor anguli oris) can be seen in Fig. 2. The data in 2 out of 20 participants were excluded from statistical analysis. During the photo task, EMG amplitude of the orbicularis oculi increased 1.13 mV on average in the mask condition relative to the no mask condition and this increase was statistically significant (V = 168, n = 18, corrected *p* < 0.001, r = 0.847). In contrast, the difference between the no mask and mask conditions was not significant for either zygomaticus major (0.61 mV; V = 117, n = 18, uncorrected *p* = 0.182, r = 0.323) and depressor anguli oris (1.95 mV; V = 138, n = 18, uncorrected *p* = 0.021, r = 0.539).

**Figure 3:**
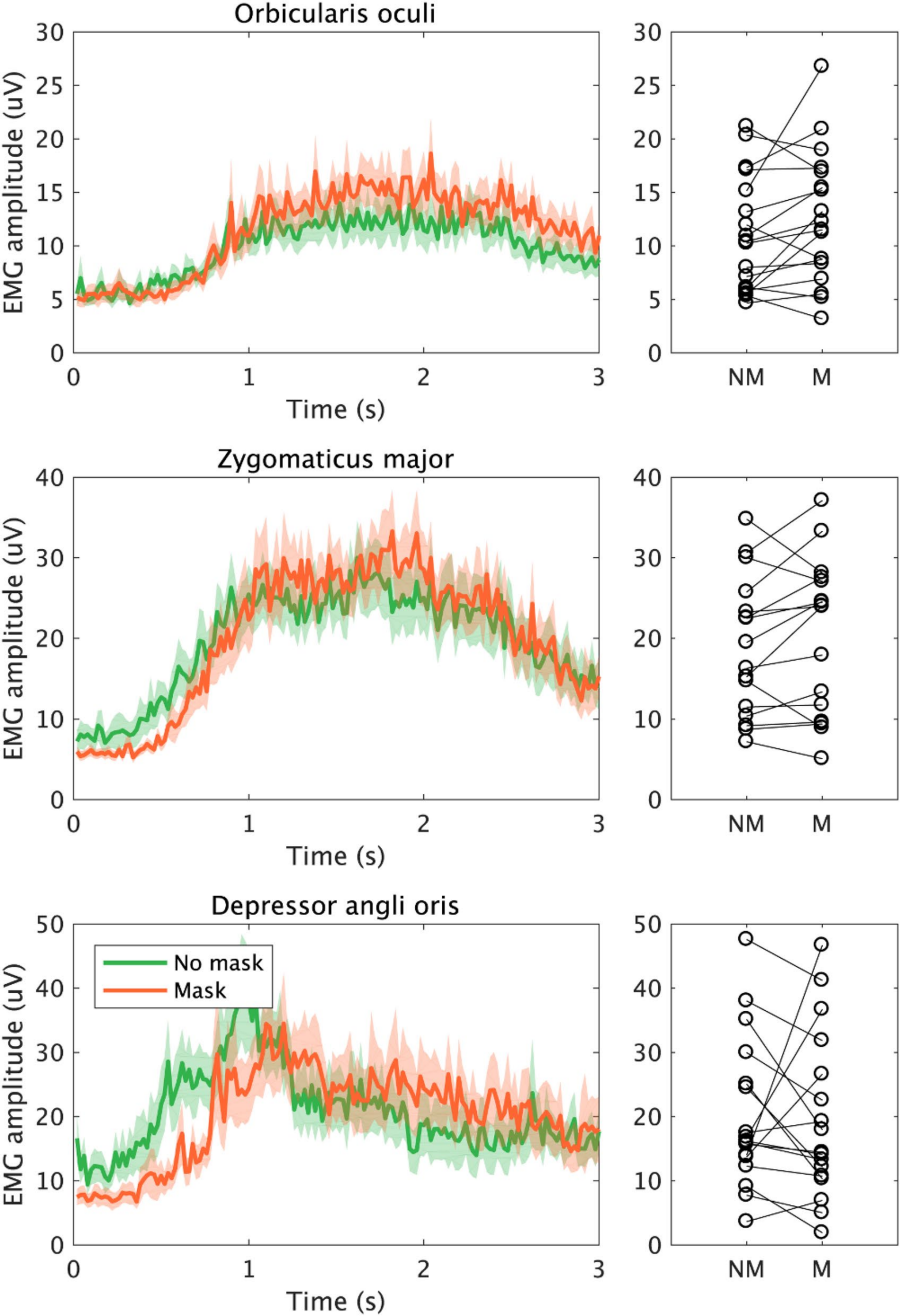
EMG signal measured over orbicularis oculi, zygomaticus major, and depressor anguli oris, during the smiling task (3 s). Plots and colours are the same as in Fig. 2. There were no significant changes in the muscle activity of the three muscles in the smiling task.

The smiling task group average EMG signals can be seen in Fig. 3. 2 participants were excluded from the analysis. For smiling task, the amplitude of the orbicularis oculi and zygomaticus major both increased by wearing mask although neither were statistically significant (orbicularis oculi: 1.65 mV, V = 123, n = 18, uncorrected *p* = 0.108, r = 0.385. Zygomaticus major: 1.32 mV, V = 114, n = 18, uncorrected *p* = 0.228, r = 0.293; respectively). The amplitude of the depressor anguli oris decreased by wearing the mask but this was not significant (-0.34 mV, V = 67, n = 18, uncorrected *p* = 0.442, r =  − 0.190).

**Figure 4:**
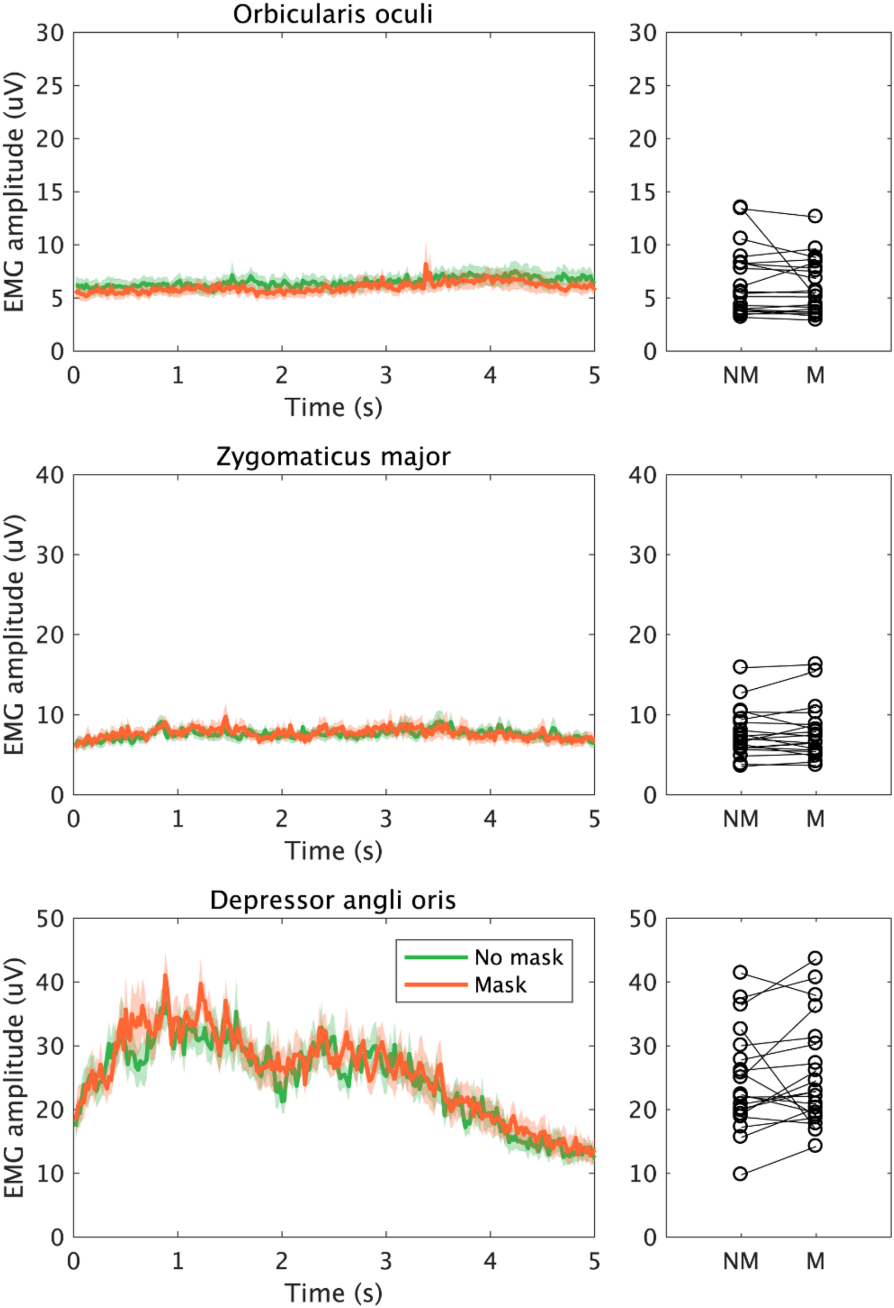
EMG signal measured over orbicularis oculi, zygomaticus major, and depressor anguli oris, during the reading task (5 s). Plots and colours are the same as in Fig. 2. There were no significant changes in the muscle activity of the three muscles in the reading task.

The reading task average EMG signals can be seen in Fig. 4. There was a decrease in average EMG amplitude of orbicularis oculi of 0.49 mV by wearing mask which was not statistically significant (V = 77, n = 20, uncorrected *p* = 0.312, r =  − 0.234). Zygomaticus major showed a nonsignificant increase (0.10 mV, V = 118, n = 20, uncorrected *p* = 0.648, r = 0.109) as did depressor anguli oris (1.12 mV, V = 146, n = 20, uncorrected *p* = 0.133, r =  − 0.342).

**Figure 5:**
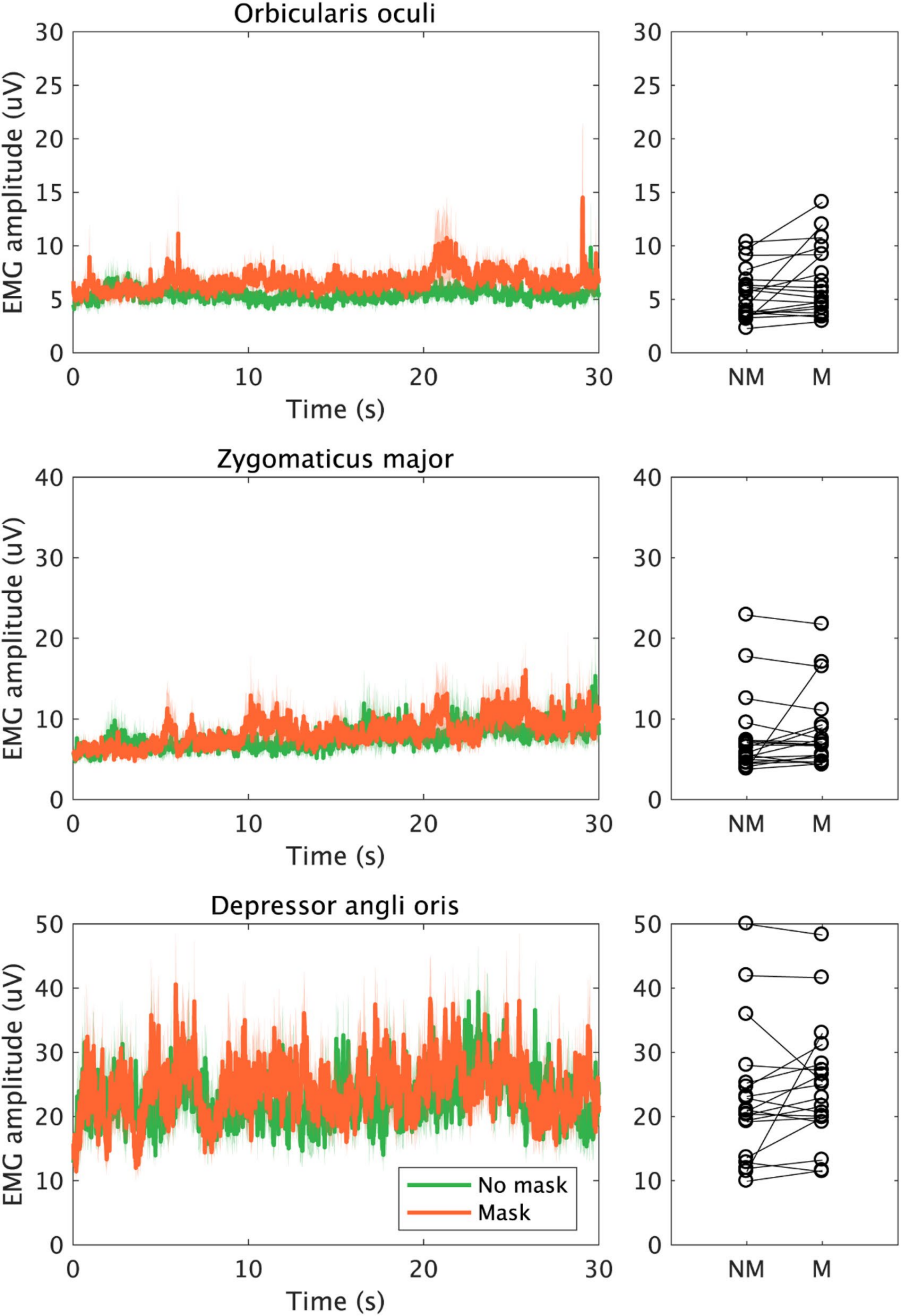
EMG signal measured over orbicularis oculi, zygomaticus major, and depressor anguli oris, during the talking task (30 s). Plots and colours are the same as in Fig. 2.

The talking task average EMG signals can be seen in Fig. 5. 1 participant was excluded from the analysis. The averaged EMG amplitude showed a non-significant increase for orbicularis oculi (1.22 mV, V = 146, n = 19, uncorrected *p* = 0.040, r =  − 0.471), zygomaticus major (0.85 mV, V = 109, n = 19, uncorrected *p* = 0.595, r = 0.129) and depressor anguli oris (1.81 mV, V = 129, n = 19, uncorrected *p* = 0.182, r = 0.314).

## Discussion

During the coronavirus pandemic, wearing a mask has become common and is obligatory in some places. As such, there has been increased interest in the various effects of mask-wearing, including the possible interference effect the mask has on communication of emotion via facial expression. Concerns regarding possible interference may have exacerbated the resistance to mask-wearing seen in a number of countries. Overcoming the interference during wearing the mask is thus an important goal. As mask wearing blocks the transmission of emotional information from the lower face, we hypothesized that wearing a face mask induces a compensatory increase in the action of muscles around the eye that indicate the individual is smiling.

We tested this hypothesis by measuring face muscle activity using EMG. Specifically, we looked at the pattern of change in the activity of orbicularis oculi, zygomaticus major, and depressor anguli oris while smiling during mask-wearing. We found that eye involvement in smiling (as measured by orbicularis oculi activity) was enhanced when smiling for a photograph during mask-wearing compared to no mask (Fig. 2). In contrast, the muscles related to the mouth (zygomaticus major) and speaking (depressor anguli oris) showed no change between the masked and mask free condition when they made a volitional smile as they would while being photographed or when asked to smile (Figs. 2, 3). Similarly, no mask related change was seen in these two muscles when individuals spoke (Figs. 4, 5). Thus, there was sometimes an adaption in the use of the eyes to indicate a smile, but this did not change the behavior of the muscles creating a smile with the mouth.

Recent studies have demonstrated that the emotion of the person wearing a facial mask is less accurately recognized^5,6^. These findings focus on how the mask wearer is perceived, while our study focuses on how the behavior of a mask wearer is modified during communication while wearing a mask. The Duchenne smile was previously thought to be indicative of a person really feeling enjoyment^8,17^. However, recent studies have argued that eye involvement during smiling can be intentionally controlled even in the absence of enjoyment. As a consequence, it is not clear in the current study if the increase activity in orbicularis oculi was involuntary or voluntary.

Although we found a significant difference in EMG activity around the eye during the photo task (Fig. 2), no difference was found the smiling task (Fig. 3). Krumhuber and colleagues have argued that the occurrence of Duchenne smile is associated with the spontaneity of the expression^18^. As smiling in a task in response to a beep stimulus with no person watching is less spontaneous than the photo task, the task difference in the results may be due to the difference in spontaneity. Their appearance in the photo task movie may also have activated automatic non-verbal communication processes, which would not have occurred in response to an auditory cue to smile.

There are two limitations of this study. The first is that the participants in this study were all Japanese females. As noted before, mask culture in Japan has survived for a very long time^1^. It may be the case that through long exposure to the existing mask culture, Japanese participants have developed flexible control over the involvement of their eyes during smiling. This may not be the situation in countries where there is no mask culture. In addition, increased control over eye involvement in smiling may also result from the cultural tendency in Japan (and other East Asian countries) to derive emotional information more from the eyes than the mouth, which is the opposite of Western cultures^19–21^. This interesting point can be addressed in a future cross-cultural study and clarify whether the effect observed in this study is generalized to non-Japanese females populations.

The second limitation is we did not measure whether the adaptation in response to the mask was effective for communication. That is, we did not measure whether others could perceive the effect produced by increased orbicularis oculi muscle activity when smiling with the eyes while wearing a mask. It thus remains unclear to what extent the enhancement of the eye involvement in smiling seen in this study compensates for the hidden mouth. Previous studies suggest that masks do impair recognition of emotion^5,6^, but this interference effect may be overestimated because these previous studies used photographs where the facial expressions were masked using a digitally added mask. Therefore, there would have been no eye compensation in the mask stimuli. It may be the case that the enhanced eye involvement in smiling seen in the current study is enough to compensate, especially if it occurs together with the enhancement in gestures^22^ and prosody^23^ that might be expected in a natural setting.

## Conclusion

Eye involvement during smiling can be enhanced when people communicate their smile while wearing a face mask compared to without a mask. If confirmation of the effectiveness of this enhancement and replication in a setting of increased ecological validity can be obtained, concerns about mask interference with emotional communication may be alleviated.

## Data Availability

The datasets generated during and/or analysed during the current study are available from the corresponding author on reasonable request.
